# American Dental Convention

**Published:** 1860-09

**Authors:** 


### Proceedings of Societies.

### AMERICAN DENTAL CONVENTION.

The sixth annual session of the American Dental Convention was held at the Metropolitan Hall, at Saratoga, on Tuesday, Wednesday, Thursday, and Friday, August 7th, 8th, 9th and 10th, 1860, commencing on Tuesday, the 7th, at 10 o'clock, A. M.

The Minutes of the last meeting were read and approved, after which those desiring to become members enrolled their names and paid the annual dues.

### LIST OF MEMBERS.

*New Hampshire*—P. A. Stackpole, Dover.

*Vermont*— M. Tefft, W. Poultney; H. Kingsley, Middlebury; E. V. N. Harwood, Rutland; D. W. Prime, Brandon; L. Gilman, St. Albans; D. W. Prune, Brandon; A. P. Hall, Rutland.

*Massachusetts*—I. J. Wetherbee, Boston; L. A. Cox, Pittsfield; C. S. Hurlburt, Springfield; S. G. Henry, Westboro'; Thos. Palmer, Fitchburg; F. Searl, Springfield; W. L. Bowdoin, Salem; J. M. Gregory, Southbridge; George T. Cook, Milford.

*Connecticut*—R. G. Reynolds, Waterbury; Saml. Mallet, New Haven; W. W. Sheffield, New London, W. Potter, Norwich.

*New York*—A. Westcott, Syracuse; D. Vandenburgh, Oswego; A. McIlroy, S. Covell, New York City; A. N. Priest, Utica; T. H. Burras, New York; P. Hogan, Waterford; E. F. Wellson, Rochester; B. S. Brown, Buffalo; J. C. Gifford, Westfield; W. B. Hurd, Williamsburgh; B. W. Franklin, New York; D. S. Golday, Oswego; R. G. Snow, B. T. Whitney, Buffalo; S. W. Robinson, Watertown; S. P. Arnold, Ballston

Spa; J. S. Latimer, New York; Thomas D. Evans, New York Mills; J. C. Munroe, Brooklyn; E. A. L. Roberts, A. Merriman, W. B. Roberts, F. H. Norton, George H. Perine, New York; S. L. Smith, Clayville; W. H. Dwinelle, John Allen, New York; M. W. Wilson, Saratoga; F. E. Parkman, Troy; S. B. Palmer, Tully; Geo. E. Knox, Glenn's Falls; Jos. Chandler, Syracuse; Jas. C. Duell, Amsterdam; A. C. Clark, Poughkeepsie; George Clay, New York; J. P. Beardsly, Clinton; E. Beckwith, Westmoreland; D. Knower, Schoharie; S. W. Thurston, Fort Edward; John E. Savery, Cayuga; W. H. Clark, Little Falls; N. W. Shattuck, Troy; J. S. Dodge, jr., New York; J. W. Tefft, Ticonderoga; J. C. Austin, Albany; Wm. Dalrymple, New York; W. L. Lee, Sodus; J. A. Perkins, Albany; C. L. Reik, Schuylerville; Chas. S. Niles, J. D. Chevalier, New York; H. Jameson, jr., Lyons; A. C. Haws, New York; L. W. Rogers, Utica.

*New Jersey*—G. T. J. Colburn, Newark.

*Pennsylvania*—S. S. White, Philadelphia; C. B. McDowall, Comeansville; T. L. Buckingham, J. R. McCurdy, J. M. Asay, J. F. Asay, Philadelphia.

*Virginia*—James Johnson, Staunton.

*South Carolina*—B. A. Rodrigues, Charleston.

*Georgia*—F. Y. Clark, Savannah; H. B. Arnold, Thomasville; W. Johnston, Savannah.

*Louisiana*—D. Bentley, Ponchatoula.

*Tennessee*—E. D. Wheeler, Murfreesboro'; C. F. Münter, Jackson; W. H. Morgan, Nashville.

*Kentucky*—A. S. Talbert, Lexington.

*Ohio*—W. A. Pease, Dayton; J. Taft, Cincinnati; W. H. Atkinson, Cleveland.

*Long Island*—S. W. Sutton, Green Point.

*Florida*—P. P. Lewis, Tallahasse.

*District of Columbia*—M. Loomis, Washington.

*Illinois*—W. W. Allport, Chicago.

*France*—C. S. Putnam, Paris.

*England*—E. Gidney, Manchester.

The Executive Committee was called, and presented the following report, viz:

1st. Reading Minutes of last meeting.
2d. Reports of Officers and Committees.
3d. Admission of Members.
4th. Election of Officers.
5th. Retiring President's address.
6th. Induction of Officers elect.
7th. Miscellaneous Business.
8th. Essays and Discussions.

All Essays shall be read to open the discussions on the subjects to which they relate, and they shall be limited to 20 minutes in length.

I. ARTIFICIAL DENTURES.
Preparing the Mouth.
Materials for and modes of obtaining impressions and models.
Various Bases and their Manipulations.
*Metals.*
*Minerals.*
*Gums.*

II. STRUCTURE AND NUTRITION OF THE TEETH.

III. IRREGULARITIES. *Causes.*
*Treatment.*—Prophylactic, and Remedial.

IV. SENSITIVE DENTINE. *Pathology.*
*Cause.*
*Treatment.*

V. EXPOSED OR WOUNDED PULP.
*Prognosis.*
*Treatment.*—Preservative, and when devitalized.

VI. DISEASED DENTINE.
*Pathology.*
*Causes.*—Remote and proximal, including the effect of different diseases and medicaments upon the teeth.
*Treatment.*—Prophylactic, *general and local.*
Remedial, *general and local*, including materials for and modes of filling.

N. B.—No member to speak more than fifteen minutes or more than twice upon the same subject without permission.

9th. Exhibitions of Models, Improvements or Inventions, with Miscellaneous Discussions or unfinished business.

| | |
|---|---|
| GEO. F. FOOTE,<br>JOSEPH RICHARDSON,<br>T. H. BURRAS,<br>JEREMIAH MASON,<br>S. W. ROBINSON, | Executive Committee. |

The Chair announced that the election of Officers was next in order.

The Chair announced Drs. Burras, Rodrigues and Searl as tellers. A number of candidates were nominated and balloted for, when it was found that Dr. T L. Buckingham, of Philadelphia, was elected President: Dr. B. A. Rodrigues, of Charleston, S. C., Vice President; Dr. B. W. Franklin, of N. York, Recording Secretary; Dr. I. J. Wetherbee, of Boston, Corresponding Secretary; Dr. A. N. Priest, of Utica, Treasurer.

The Executive Committee for the ensuing year consists of Drs. F. Y. Clark, Savannah, Ga.; F. Searl, Springfield, Mass.; B. T. Whitney, Buffalo, N. Y.; B. D. Wheeler, Murfreesboro, Tenn.; and Wm. A. Pease, Dayton, O.

The retiring President made the following remarks:

RETIRING PRESIDENT'S ADDRESS.

*Gentlemen of the Convention:*—The next thing in the order of business, as arranged by your Committee, is an address by the retiring President. I should have been well pleased if your Committee had also given me a subject. As it is, there is no particular question before the House, and we will, if you please, consider ourselves in "Committee of the Whole, on the State of the——" *profession.*

I hope gentlemen will not be alarmed at the allusion; I do not intend to make a long speech, but on the contrary a very short one. I therefore protest against your leaving the house or reclining your heads for a nap, for I shall be done almost before you could get your liquor or compose yourselves to sleep.

When we look at Dentistry as it is, and compare it with what it was a few years ago, we are astonished at the progress that has been made. There is probably no art or science in which there has been such great advancement in the same length of time. And this change is seen not only in what has been attained in the principles and practice of the profession, but also in the character and standing of its members.

It is but a short time since all that was known of dentistry was thought to require no higher talent or skill than that of a village barber or blacksmith. But it has now risen to the dignity of a respectable profession, which, to be successfully followed, demands long study and thorough training, with an amount of scientific knowledge and practical skill equal to that of any other profession.

It must be confessed, however, that many who "profess and call themselves" dentists are sadly deficient in the qualities and qualifications mentioned. We have able writers—learned professors and skillful operators; with, perhaps, a fair proportion of members of respectable talents and attainments, but there is still among us a large class of mere *tooth-tinkers*, who are scarcely worthy to rank with the barbers and blacksmiths of former days.

It is true that quacks and pretenders are common to all professions and avocations, but they are more degrading to dentistry, because the public has no certain means of distinguishing between the worthy and the unworthy.

The swarms of charlatans that hang around other professions are known to be outsiders, with no title to stand among *regular* practitioners. But any man may open an office and call himself a dentist. No diploma, license or certificate is necessary to make him just as regular as the best. There is among us no recognized authority to declare who are and who are not qualified to practice, nor have we any certain rule by which that qualification could be determined. If we were to require a regular course of study, it would exclude many of our best dentists, who commenced practice when there were no adequate means of obtaining a dental education. In thus attempting to pull up the tares, we should pull up the wheat also.

This is a state of things incidental to a new profession. The demand for dental service in this country opened a wide and almost unexplored field of investigation, which, with its promised emoluments, attracted great numbers into the practice. Among these were many ingenious, inquisitive men, who, untrammeled by precedents, and unawed by authority, pursued their experiments with a freedom and boldness, not always indeed to the advantage of their patients, but resulting on the whole in valuable improvements and discoveries.

These, and such as these have contributed much to make dentistry what it now is. But they have been succeeded by a horde of impostors who are a disgrace to the profession.

Is there no way of cutting them off? Must we always submit to such associations? I would not be arrogant or exclusive. It may be desirable, it is certainly inevitable that there should be a wide difference in the standing and attainments of dentists. I would recognize the humblest degree of skill that can be honestly useful among any class of people. But to be obliged to stand among impudent knaves and charlatans, with nothing to distinguish us from them, is too humiliating to be endured.

It is a legal maxim that where there is a wrong there is a remedy. Perhaps, in this case it is difficult to find the remedy, or to apply it when found. Our condition is anomalous. We are standing on the body we wish to move.

Well, gentlemen, I have nothing new to recommend. The only remedy for the evil, which occurs to me has been often proposed. It was particularly insisted upon in a letter to the New York State Dental Association, written by Dr. Westcott last September. It is in substance this:

That a number of our most prominent dentists in this State (and the same may be done in other States) should obtain a charter from the legislature for a Dental Institute, with power to hold property, establish a library, provide lectures, etc., and to grant diplomas to such persons as after due examination should be found qualified to practice.

This plan may not be without objections, but it seems to be the only one that is now practicable; and I am sure it could be carried into effect if the leading members of the profession would give it their support. But precisely here is the difficulty. Many of our most eminent dentists persistently stand aloof from all organizations for fear of being associated with the class of persons of whom I have spoken.

This is wrong. It is selfish. It is in their power and it is their duty to do something to purify and elevate the profession to which they belong. In refusing to do this, they illustrate the virtue of those politicians who profess to be for "the greatest good of the greatest number," but with whom the greatest number is always *number one.*

Let these men step down from their lofty pedestals and lend a helping hand to their laboring brethren, and they will soon find themselves belonging to a profession of which they will have no cause to be ashamed.

As I promised to be short, I will not now pursue this subject farther. Much has been said of late about "the elevation of our profession," and I have suggested one of the readiest means of doing something towards that end. But after all, we must not expect too much from this or any other organized effort. I said the evils of which we complain are incidental to a new profession. We must be patient and wait for the operation of causes that will slowly but surely bring about a change.

Some have looked to this convention to be the great winnowing machine that should separate the chaff from the wheat. But it never had any such purpose or design. If there was any great danger of our being overrun by unworthy persons, we could adopt some restrictions for our own protection. But we have so far had no cause to complain.

Permit me to say, in closing, that in reference to the elevation of our profession, there has, in my judgment, been too much of boasting and self-glorification among us. Some have been too forward in *pressing* the claims of dentistry rather than wait for them to be recognized by the community. No person or profession ever attained a permanent standing by talking big or putting on airs. The best way to secure respect is to deserve it, and the surest way of elevating our profession is for us to individually elevate ourselves.

It only remains for me to thank you, gentlemen, for repeated expressions of your confidence and esteem, and to assure you that after service in office for three successive years, I now retire, with unabated interest in the objects of this convention.

Drs. ALLPORT and DWINELL were appointed to conduct the President elect to the chair.

On motion, a vote of thanks was tendered to the retiring officers.

On motion, it was

*Resolved*, That this convention hold its sessions as follows:—

Meet at 9 a.m. adjourn at 1 p.m.
" 4 p.m. " 7 p.m.

The new President was then conducted to the chair, and after a few remarks from him the Convention took a recess to 4 o'clock.

### 4 O'CLOCK P.M.

The Convention met according to adjournment. The President in the Chair. The regular order of business was taken up, viz: " Artificial Dentures."

Dr. B. W. FRANKLIN read an interesting and instructive paper upon Mechanical Dentistry. (Which will be published—Rep.)

Dr. JOHN ALLEN, of New York, also read a paper on Mechanical Dentistry, upon the structure of artificial dentures,

(which is published in full in this number of the Register.—Rep.)

On motion, it was

*Resolved*, That no person who has not signed the roll and paid $2 00 shall be considered a member of this Convention; neither shall his name appear in the printed proceedings.

Dr. F. Y. CLARK was called upon, and read a paper upon the subject of obtaining metallic dies from the plaster impression; also made some oral remarks on the subject. He observed, that he generally used for the impressions a batter composed of equal parts of plaster and spar, sometimes changing the proportions to plaster one part, and spar two parts; take the impression as usual. Uses a perforated impression cup. Place the impression in flask No. 1, of his series, filling up the flask around it with the plaster and spar; then place on the body of the flask; dry it on the fire, then pour in the metal—he prefers zinc; then turn the flask; remove the plaster, and pour in the lead; then you have the model and counter model. By this method you obtain the most perfect model; a slight contraction of metal is advantageous, thus the use of moulding sand is avoided; absolute dryness of the impression is not necessary, yet the dryer the better. This batter is better than anything else for taking impressions for partial sets. The natural teeth draw well from it. It has more elasticity than plaster alone.

Dr. FRANKLIN first takes impressions in wax, trims out of uniform thickness, about a line in depth, all over the impression. The advantages of this method are, that many of the defects attendant upon the ordinary method are obviated, such as perforations or bubbles; there will be far less plaster in the mouth, and less liability of it running into the throat. There will be less expansion in setting and drying than if a larger amount of plaster was used.

A plaster model will not change, he thinks, under a pressure of steam of 600°

Dr. BURRAS procures an electrotype from the plaster impression. Having a correct impression, cut it down to the line to which the edge of the plate must come; cut out a chamber; varnish the model over, except that part where the plate is to be, that part blacken with plumbago. Electrotype the *impression*, in this electrotype pour britannia, and thus make the model.

Dr. Westcott remarked that we sometimes think we have made an invention when we have not; thinks that Dr. Burras' plan is one of the most beautiful things in the world, the only objection is that too much time is consumed; the more expedition we can attain the better, this is a fast age, slow things will not answer.

Dr. W. B. Roberts.—Electrotyping is not a new invention, if Dr. Burras has been using this process, does he use it all the time? Thinks it is not good; the deposit is soft as lead, and will batter down. Why have they not published it before? Is it not just a fancy operation, presented for effect?

Dr. Westcott.—The copper is not soft, it is hard enough for practical purposes, the copper faced type is an evidence of this.

Dr. Burras admits that the process is somewhat tedious, but then maintains that the advantage to be gained is more valuable than the time employed in the process.

Dr. W. B. Roberts.—The time required for electrotyping is greater than most people suppose; it is an intricate process, and one that but few can or will be familiar with; the deposit may be made with various degrees of density.

Dr. Sutton thinks Dr. Clark's method a very good one, and will supercede the electrotyping process; has used the same principle as Dr. Clark for some time; used plaster and spar for impressions nine years ago; dried very perfectly. Drills holes into the impressions from the underside, almost through the plaster, so that the vapor may freely escape; uses pure zinc for models, and lead for counter models; finds nothing better upon the whole.

Dr. WETHERBEE—the plan here presented by Dr. Clark is in every respect feasible; the contraction does not amount to anything; thinks it much better than those more complicated methods.

Dr. ATKINSON remarked that the profession has been in the habit of waiting too long after extracting the teeth; his method is, after extracting the teeth and stopping the hemorrhage, to trim the gums and process; constructs a temporary set and inserts at once; trim slightly in hard mouths and extensively in soft ones; trim as near the supposed permanent form of the gum as possible. For trimming the gum and process uses the scissors and bone forceps.

W. B. ROBERTS—we often suppose we have a new idea, but find out that some one has preceeded us; he suggested the following, knows it to be good. When three, four, or five teeth remain, take the impression; strike up plate, fit well, cut out round the teeth, articulate and arrange the teeth, same as for a partial set; cut off the plaster teeth and fit the artificial teeth; (the impression is taken before extracting.) Does not adopt Atkinson's method, although a good one, as it will sometimes fail; the rule is to give as little pain as possible; had some experience, therefore can sympathize. If you can take impressions and fit plate same day the teeth are extracted, it is all the better; the plate protects the parts, and prevents pain during mastication, if you run the plate over the sore places; the absorption does not take place; cut the plate out in that part, let the teeth fit over the sockets, depending on the palatine arch for support. There is no rule to tell when absorption is complete. Some will take six months, some six years.

Dr. WETHERBEE thinks this discipline of great importance to the profession. Thinks great mischief occurs partly from the patient wanting the artificial teeth immediately. Three fourths, of his own cases, he is satisfied are put in too soon. Often where there are protuberances, takes a cutting forcep, clips off the prominent parts of the process, and with a pair of scissors clips off the fragments of gum. Adopting this *modus operandi*, secures, in young patients, what cannot be by other means. The mode is to add springs to the plate; in this way the same plate is worn until absorption is complete, with equal pressure—no toppling to the one side or to the other. Two years ago made similar remarks—cut the cast much more than the mouth.

Adjourned to meet at 9 o'clock to morrow morning.

SECOND DAY—9 A. M.

The Convention met according to adjournment.

The minutes were read and approved.

Same subject continued.

Dr. JOHN ALLEN'S practice is to extract the teeth, then insert the artificial denture, within a week or two; does not trim the gum or process, but crumbles off the sharp points of the process. The absorption goes on as well under the plate, as without it; usually trims down the plate at the sharp points. Uses continuous gum invariably; does not find it necessary to make a second set. Various reasons why the set should be put in immediately after extraction, the patient the more readily accommodates himself to them, than when left too long.

Dr. WHITNEY extracts with great care, but never cuts down the process, or gum; endeavors to get the substitutes in the same day of extraction, the parts are more readily accustomed to them; objects to claiming permanency for the teeth substituted; gives no such promise to the patient. As long as they can be worn with comfort it is well. Suggests that we forget the name *permanency*.

Dr. SEARL thought rather too much time was being consumed on this subject, and moved that we now proceed to the next subject.

After some discussion the question was withdrawn.

Dr. COVELL objects to the use of vulcanite base because of its offensive odor; also maintains that the sulphur will be injurious to many persons, as is well known. The rubber will absorb the saliva more than porcelain, and thus become offensive. His method of inserting artificial teeth is different from that of many others; he does not use chambers; avoids vacuums in *all* cases if possible; thinks it objectionable; his plates set better than the plates of those who use vacuums. He would not wear a plate as Dr. Wetherbee inserts them, and would not ask his patients to wear them thinks they would not endure it.

Dr. HURD—we have different ways of expressing ourselves—do not think that the sulphur in the vulcanite base will injure any one, there certainly is not enough sulphur for this. Each one obtains his results from his own experience and circumstances. In inserting artificial dentures, there is great diversity of results with different kinds of patients. The dentist who does his duty to his patients will find that he has more to contend with than any other profession. In preparing the mouth, extract the teeth as carefully as possible without lacerating the gums; disturb the parts as little as possible.

Dr. GOLDEN thinks that absorption will take place where the greatest pressure is made. Thinks that sulphur in the rubber work will not injure any one.

Dr. WETHERBEE.—Dr. Allen's method is next best to his own. In Dr. A's plan the patient cannot wear the temporary teeth as long as in his own method. It is said there is no such thing as a permanent set of teeth, this he denies; has inserted sets of teeth ten years ago, that are as good as when they were put in.

Dr. ASAY—what does Dr. Wetherbee mean by temporary teeth?

Dr. FRANKLIN—we should not attack the modes and plans of others, but state those of our own.

Dr. ASAY—what do we understand by a temporary set of teeth? When a patient is presented, he considers all the circumstances, the health and age of the patient; extracts the teeth, and if a patient desires it, inserts a set of teeth, calls it a permanent set of teeth, and if it has to be remodeled charges for it.

Dr. FRANKLIN has spoken more in favor of other kinds of work, than of the vulcanite; if he was in full practice should use all kinds of work; chooses to refer to the principles as well as the manipulations. The chemist cannot find a trace of sulphur in vulcanized rubber. Rubber base will not absorb saliva more than any other material employed. The material requires to be properly and carefully manipulated, then it can be properly and carefully finished, and is permanent enough for good work.

Dr. ROBERTS will give a few facts in regard to mounting teeth; have used all the kinds of work employed; used vulcanite a year before Dr. Franklin did so. It is said that rubber has some advantages that other modes have not, for instance, that it is easily made and fits more perfectly than anything else, and that it is valuable for permanent work. The rubber is prepared, with rubber, sulphur and oxyd of mercury; thinks this is objectionable in regard to the material. The continuous gum work is easier mended than vulcanite; it, is *easily* mended—the rubber work is difficult to mend because it changes shape and changes color.

Dr. TAFT remarked that all styles of work were valuable; each one has its merits and demerits, one is better adapted for a certain case than anything else, and professional wisdom enables the dentist to select that mode which is best in any given case. He confined his remarks chiefly to continuous gum work, and to one special application of that kind of work, viz: the construction of temporary sets of teeth. The plan of proceedure is as follows: extract the teeth; if the gums are rough trim them with the knife and scissors, if there are sharp points of process cut these off also; then take the impression at once or within a few days; prepare the casts in the ordinary manner, and form a plate from thin plate No. 30 to 34 Stubb's guage, let the plate extend a little outside of the middle of the ridge; then attach continuous gum teeth properly selected, as usual; make the antagonism as perfect with the opposite teeth as possible. For putting on the body, set the piece on the plaster model, then put on the body round the necks of the teeth, trimming the margin neatly, and packing it on to the inequalities of the model representing the gum from which the teeth were extracted, the body thus fits this perfectly—care should be exercised to give just the natural fulness. The piece is then biscuited, and tried on the cast, or into the mouth if necessary; in putting on the second body, any little deficiencies should be remedied. When the piece is finished it fits perfectly the inequalities of the ridge, and no one not acquainted with the fact would detect them as artificial teeth. The gum will, after a time, absorb away and leave a space above the border of the piece all round; when this is accomplished, so as to interfere with the utility or appearance of the piece, then an accurate impression should be obtained, and a model from this; this model should consist of equal parts of plaster and spar. Place the denture on this model; now build n body to compensate for the absorption of the gum and process, attaching it to the plate and body and adapting perfectly to the model; then remove it if desirable; try it n the mouth; then put on the second body and gum, and finish. The over-lapping portion should extend sufficiently ar up that the edge will not be exposed when the mouth is open in speaking. There is, of course, no plate beneath this projecting portion, but if properly manipulated it is sufficiently strong. There are several advantages attending this method. The patient has useful teeth immediately after osing the natural ones; does not loose the use of teeth; it effectually conceals the presence of artificial substitutes. The form of the mouth and face is far more perfectly retained, during the absorption, than if teeth were not worn; the muscles are not subjected to the same amount of contraction. Others might be named, but let these suffice.

Some of these points may be attained in the use of other styles of work, but by no other mode can they all be obtained.

The subject of Mechanical Dentistry is a large and interesting field for discussion.

On motion—

*Resolved,* That the discussions of the subject close at 12 M. to-day.

Dr. ATKINSON presented a model from Dr. Palmer, of Warren, Ohio, exhibiting the method of carving teeth in blocks or single teeth, on a model. It consists of carving the whole in one, two or three pieces, and then cutting them into as many pieces as desirable, extra amount of grinding and fitting is thus avoided.

Dr. CLARK referred to a difficulty that he had met with in inserting a set of teeth, the patient could not draw up the plate; fitted the plate as well as possible, made a Cleveland chamber but it would not work, then made another plate, with the common chamber, and swaged the plate; just back of the chamber, within a line of its border made a hole; the patient could then draw the plate up firmly; closed up this hole, and made three others through the plate, just back of the chamber. Then the patient could draw the plate firmly in place and it has been worn several years, with the very best results, giving entire satisfaction. Cutting the plate away would answer the same purpose, he supposed.

Dr. COLBURN—has used nothing but continuous work for eight years; he thinks nothing else is so good. There are less accidents with it than with gold work, and it is easier mended. Thinks it should supercede everything else.

Dr. MERRIMAN—formerly extended the plate back as far as possible, now does not do so. Makes the plate short.

Dr. GILDEA uses platina and arsenic for a solder for continuous gum work; there have been objections made to it. Fit up for soldering as usual; take small pieces of solder and put on the joints, put silax on round the point to be soldered, to prevent the solder from spreading over the plate.

Dr. Searl—is in favor of gold work with single or block teeth, thinks that new plans should be cautiously employed; has not employed other styles of work to any considerable extent, but always had trouble when he attempted them.

Dr. Allen—used the platinum and arsenic solder formerly, but abandoned it because it made the plate brittle, and flowed over it too much; now uses a solder of platinum and gold, with a slight addition of arsenic.

Dr. Clark (of Poughkeepsie)—has not used temporary sets of teeth for some time. After the teeth have been removed for two months, put up the temporary teeth, take a *thin* platinum plate and put it up so that the plate will conform to the mouth. Generally prefer gold for temporary work, do not think that rubber work is good for partial sets.

Dr. Clay—after taking out the teeth, trims the gums, and on the cast fill up the sockets; generally covers the gums in front with the plate; puts in permanent teeth in a year after the teeth are removed; used plate No. 38 permanent work; puts a wire on the ridge from heel to heel of the plate; solders only at each end to the heels of the plate; all the intervening portion of the wire is not attached to the plate. Use the single pin teeth, bend the pins to the wire and solder them to it instead of the plate; this is done that the position of the teeth may be changed, not being attached to the plate; perforate the plate with many small holes, that the body may run through for attachment. Makes light work; uses but one plate.

Dr. Wescott—he agrees that the sooner a plate is inserted the better; this produces a rough ridge, however, prefers to keep the ridge smooth. After the plate has become loose, perforate the plate and line on the palatine side with Hill's Stopping; keep this up till the temporary plate is abandoned; may regulate the roughness by making pressure upon the prominent points; pressure will produce absorption. The sockets fill up with bone material; commonly dislikes those deep jaws or prominent ridges.

Dr. ROGERS is in favor of temporary dentures.

*Second Subject*—NUTRITION OF DENTINE.

Dr. TAFT—in regard to the nutrition of dentine, remarked, that he scarcely knew what course to pursue; he should be pleased to have the committee tell us what was intended by the language in which this subject is couched; does it have reference to the sustenance and nourishment of the teeth after development, or does it include also the development of the teeth; he would confine his remarks to the first particular, viz: nutrition of the teeth after development. The fluid, that circulates through dentine, though it does not contain all the elements of the blood, that nourishes other tissues, yet it does contain enough and is just adapted to the tissue which it is intended to nourish. The circulation of this fluid through the dentine, is not through distinct and definite vessels, as in other tissues, but is a kind of imbibition, influenced and controlled by vitality: it is also vitalized. This tooth blood is supplied both from the internal and external connection, perhaps more from the external than the internal. The relative amount of vitality received by a tooth, from its internal and external connection, is a point that is not very definitely settled as yet. The teeth in a good constitution, and under favorable circumstances, assume a condition corresponding to that of the general system; and with a deteriorated state of the latter, the teeth suffer, so that they will not withstand the influence of injurious agents, as they would otherwise do. The teeth are nourished in the same manner as cartilaginous tissues are; the red globules do not pass into these, neither do they possess definite circulatory vessels. This subject is one that has received but little consideration, at the hands of our profession, though it is a very important one.

Dr. ATKINSON remarks that we have first plasma, then cells, then tissues, organs and systems. The enamel represents the mineral kingdom; the dentine, the vegetable; the pulp, the animal.

What the circulation is, is indicated by the structure, where there is ossification of dentine it approached the character of the enamel.

Dr. Dodge made some remarks upon crystalography; thinks the enamel is not true crystalization. The circulation is analagous to that of other parts, it is a kind of permeation, both from the pulp and periosteum.

Dr. Wescott was formerly as fond of theories as any one, but has become more practical; the enamel is true crystal in its structure, the dentine has a circulation but not vitality sufficient to resist chemical action; vitality is the great resistant of chemical action; there is not enough vitality to resist chemical action to any appreciable extent.

Dr. Atkinson.—every tooth has its follicle when it cuts the gum; this is nourished, and that nourishment is not cut off while the vitality of the tooth remains.

Adjourned to meet at 4 o'clock, P. M.

### AFTERNOON SESSION.

Convention met according to adjournment. Same subject.

Dr. J. Allen gave the constituents of the teeth; the elements of dentine should be furnished in the food; in some kinds of food it is removed in the preparation—the preparation of wheat flour is an instance; the hull of the wheat should be left, for this contains the bone material.

Dr. Taft remarked that, as the discussion had involved the nutrition of the growing teeth, as well as those already developed, he would make a suggestion or two. This is a great subject—it is in reality the great point in this subject; it is one in which is involved not only well-being, but life itself. Probably one-fourth of all the children that die under two years of age, die from difficult dentition, or disease having an immediate connection therewith. This subject of difficult dentition and nutrition of the growing teeth, is one much more easily influenced, modified and controled, than most persons suppose. What is required is a good assimilating, appropriating and upbuilding apparatus; and the proper material to be employed. Every one should pursue that course best calculated to bring about these conditions, and then supply the proper materials. Some experiments were detailed, illustrating the importance of this subject.

Dr. SEARL—how are we to reach patients in this respect?

Dr. ALLEN—by giving the information in properly written books and circulars, diffuse the light.

Dr. BUCKINGHAM.—we lose sight of the vital forces—this must be regarded. The teeth are nourished as any other part is, notwithstanding the red globules do not pass through the dentine; the red globules do not nourish any thing. The enamel is organic and vitalized, and more resistant to chemical agents than the dentine. The enamel is not crystalline in its substance—it is molicular deposition. There *is* circulation in the dentine.

Dr. WESTCOTT—any structure containing not more than one-half per cent, of animal matter, can have but minute resistance to chemical affinity. Is it true, as just expressed, whether under the vital or vascular system, that if the body is well the teeth are well, or if the body is sick the teeth are sick? Can not admit any such doctrine, having seen very sick teeth with a well body.

If patients believe that a *live* tooth is capable of resistance, like the hand or any other part of the body, they would soon learn to let them go and take care of themselves.

Dr. STACKPOLE is highly gratified by remarks just made. Take a theory of any kind, find it may be beautiful, but when applied practically it often fails.

It is difficult to apply these theories practically, in the use of phosphate, etc., fed to the mother. Physicians, after treating a patient, often think they have cured them; are they sure of this, or did nature do the work? would they not have got well without any treatment? Believe as others have remarked, that a dead tooth in a dish is very different from a live tooth in the mouth; find often different characteristics of the teeth in different members of the same family—can we say it is hereditary? Sometimes must feel that we may be mistaken; the theory may be good, but must accord with common usage.

Dr. G. H. PERINE moved that the convention take some action in regard to assisting in raising money to erect a monument to the memory of John Hunter, sustaining the motion by remarks highly creditable. (The *Resolution* to be found in the minutes.—REP.)

Dr. GIDNEY thinks the object a laudable one. John Hunter was an ornament to the medical and dental profession, and it is highly commendable to make a contribution of this kind.

Dr. ATKINSON was against any thing of the kind—was opposed to hero-worship.

On motion of Dr. Roberts, resolved, that the motion be laid on the table.

The hour of fixing the place of the next meeting having arrived, after balloting on several locations, New Haven, Connecticut, was selected.

The convention then adjourned to meet to-morrow morning at 9 o'clock.

THIRD DAY, 9 A. M.

The convention met according to adjournment. The President being indisposed, the meeting was called to order by Dr. Rogers.

The third subject, irregularities of the teeth, was taken up.

Dr. TAFT—this is an important subject—one becoming more and more so as its treatment is more general in the profession. Irregularities are of different kinds: sometimes the root occupies the proper place in the alveolus, but the tooth is turned upon its axis, and does not present the proper surface to view upon the mouth being opened. Again, the teeth in other cases have a wrong inclination, the roots being in about the right place, but occupying a wrong inclination; thus throwing the point of the tooth out of its proper place. Again, the root of the tooth may be entirely out of its proper place in the alveolus, thus throwing the tooth wholly out of its position in the arch. This latter presents greater difficulties in the way of its correction than either of the others. There are various causes for irregularity, some of which we can in some degree comprehend. There are some, doubtless, that as yet lie beyond our ken. He then simply enumerated some of the causes, among which are a tardy development of the teeth; the slow removal of the temporary teeth; a crowded condition of the permanent with the temporary teeth; a crowded state of the permanent teeth, these being too large, all to be accommodated in the space existing for them; and a malposition of the germs in the sockets, the cause of which is some constitutional peculiarity, not well understood.

The treatment is too large a field for much discussion here; the treatment will be governed by the nature of the irregularity. If the permanent teeth are crowded with the temporary teeth, the latter sometimes require to be extracted—great caution should be exercised in this particular. When the permanent teeth are crowded, either the circle in which they stand should be expanded, or one or more of the teeth should be extracted. Any obstruction in the way of a correction should be removed. Various appliances are used in the correction of irregularities. The object is to apply force in such a manner as to bring the tooth or teeth to the proper place. Of these fixtures and their application he hoped to hear from others.

Dr. Searl don't rise to give but to receive information.

Case: Young lady twenty years old, left eye tooth half erupted; the laterals were removed by one calling himself a dentist—supposing they were the deciduous teeth. The arch is so small as to have room to accommodate but half the teeth; left eye tooth short and inclining inward, so that direct pressure can not be had.

Fitted a plate to upper jaw covering the teeth; applied clasps so as to draw it firmly to its place; the left cuspid is the one to be operated upon; patient is in a hurry, and will bear any pain he would inflict; used a spring attached to a plate, and screw to tighten as rapidly as possible.

Dr. Goldey—generally uses a bar of silver, which is held in the hand, and bite upon it; in one day the tooth is in its place, in three days the most difficult cases are overcome.

Dr. Allen has in many cases ceased to use metalic plates, but instead of it uses hard rubber, which is more easily adapted, and can be filed down as the change progresses; this is the most rapid and easy method.

Dr. Talbert—in order to secure the plate for regulating without injury to the teeth, fit the plate to the lower jaw, attaching inclined planes, to strike the teeth requiring regulation.

Dr. Evans—strikes a plate, with screws attached, to bear directly against the teeth to be pressed out.

Dr. W. B. Roberts—treatment has been pretty well handled, but no rule or time can be given, it must be left entirely to the progress of the operation. Nature made everything perfect; unless its laws have been transgressed, all teeth would be properly arranged and in perfect order, hinted at what might be the cause of irregularity. Germans, Irish and English generally have good teeth. Americans have more cases of irregularity than all others. German jaws are one-fourth wider than Americans. Irish are one-fourth longer, and so with all pure bloods. We are a mixed race, originating from different nations. You may have the narrow jaw of the Irish, and the large, broad teeth of the German, and thus they can not be otherwise than irregular.

Dr. Perine—holds in hand a cast of a case for treatment of irregularity, presented for the consideration of the members.

Dr. F. Y. Clark—had a case where the incisors pointed directly outward; the cuspids, bicuspids and molars followed, and at last stood the size of one tooth ahead of their proper position; protruding externally, what should be done?

Dr. Allen—has seen a great many cases of the kind named by Dr. Clark, and finds they are produced by sucking the thumb, and therefore calls them thumb suckers.

Dr. Covell—inquires in regard to causes of irregularity, whether it does not run in families; has under treatment one family, first the daughter, then two sons, then another daughter, both parents with good regular teeth.

Another family, first two daughters, next son, all taking on the same form of irregularity. It not unfrequently is produced by too early extraction of the deciduous teeth, permiting the arch to contract, or rather is not properly expanded. It is not only necessary to place teeth where they are wanted, but to hold them there until a new deposit of process is made to fill the space from which it has been removed; if you proceed rapidly, you must augment pain, if slowly, you must take time.

Dr. W. B. Roberts remarked in regard to irregularity running in families; thinks it is a conceded point, and recited cases to illustrate.

Dr. Asay took exception to the remarks of Dr. Roberts in regard to pure blood, etc. Acknowledges that he is at a loss to know the causes of irregularity, except in a few cases, as related by Dr. Allen, from sucking the thumb. In regard to correcting, strike up a band around the inside of the teeth of the opposite jaw from the one to be regulated, soldering on little inclined planes to strike the teeth to be forced out, and if there is any difficulty about retaining the plate, the mechanical correction will suggest itself. Under all circumstances, insist that the appliance, whatever it may be, shall be kept constantly in place, never permitting it to be removed, except as he himself removes it. Otherwise, the work is as rapidly undone as it is done.

Dr. SEARL repeated his former remarks, stating again that he in two or three days gets the tooth outside of the lower, after which it takes care of itself.

Dr. W. B. ROBERTS wished to say that he did not intend that the rule named was universal, but a general cause of irregularity.

Dr. J. A. PERKINS takes the ground that it is none of our business as to the *causes* of irregularity; we must take the cases as they come, and correct them.

Dr. COVELL spent some time in the Sandwich Islands in close observation, and found not a single case of irregularity, except where they had intermarried with Americans, store-keepers who lived on fine wheat bread, sweetmeats, etc.; also in California, also in Central America, all with perfectly formed arches, and no irregularity, so far as they came under his observation.

Dr. F. Y. CLARK, in explaining the manner of regulating the case above named, first have all the hair shaved from the head, make a metal cap to fit the back of the head, lined with a cushion, fasten to the cap an arch to reach to the condyle of the lower jaw, another to come to nearly the angle of the eye on each side, then taking an impression of the jaw, attached a band to the arms of the cap with springs to keep a constant pressure. After several days removed a bicuspid on each side, and in six weeks had the position and articulation as perfect as any he ever saw.

Dr. W. H. MORGAN has had a number of cases of a similar character, which were corrected by fitting caps to the molar teeth, and passing a spiral spring around over the labial surface of the front teeth; the effect has been accomplished in two or three weeks, but the appliances had to be continued till the bony structure was deposited, to hold them in place; in one case they were brought in three-fourths of an inch, in difficult cases it was necessary to remove a bicuspid on each side.

Dr. Covell, in his experience in the Sandwich Islands, California and Central America, found that none of the natives had decayed teeth, except those who married whites, or Americans who lived on wheat flour;—he apprehends the cause to be the absence of the phosphate of lime in wheat flour. The Sandwich Islanders live mostly on an article called poar, which contains a large proportion of phosphate of lime; they eat fish raw. Central Americans live on corn cake, made of coarsely ground corn with water.

Dr. Dodge thinks it a matter of great importance to consider causes, unless we are to be esteemed only as tooth carpenters; with all due deference to his majesty, Mr. "Phosphate of Lime," there are many other things to be considered in regard to the rude and savage. The quality and chemical constituents of the food is not the only thing; the habits of luxury, the long sleeps, etc. must be taken into consideration. As our worthy President remarked, if the hand is sick, the tooth is sick, and the whole body is sick. The teeth in their internal organization, do sympathize with the condition of the stomach, becoming more highly excited and susceptible of perceiving the touch of external substances; a nervous susceptibility is generated by diseases of the stomach and other parts of the organism; if this is so, is it not also fair to presume that it lessens the ability to resist external agents? Sometimes you find a touch upon the tooth at the margin of the gum, to produce pain, at certain times, without any signs of decay. Why is it that a female, who, having but slight specks of decay, becoming pregnant, loses all her teeth during a single period of gestation, and especially if she suffers much in her pregnancy?

The proper nutrition is drawn away from its proper course, to give nourishment to the fœtus.

Dr. Rogers some time ago commenced a series of experiments of filling teeth with materials other than gold, then finishing with gold; soon found that it would not do to use any other than one metal; his theory was, that if the metals were kept free from moisture, there could be no galvanic action, but found the facts to be otherwise, in the destruction of both teeth and material. Then tried gutta percha, filling cavities nearly full, then finishing off with gold, and can now, after several years' experimenting, see no external differences between these and his best gold filling—to be sure, has taken out some filling, and found the inside white, or whiter than is usual in all gold. Another practice has been to cleanse out a large cavity, then take a piece of gold plate, bending the corners so as to form feet. Place it in the cavity, to form a stool, leaving all empty beneath, and filling with gold on this stool; in removing filling, found the feet of the stool imbedded in new dentine. It is a matter of great importance if we can leave a vacuum, with the mouth hermetically sealed, and secure the deposit of new dentine, we will have gained a great deal. Has many other cases, which will be opened, examined and reported in due time.

Dr. W. H. Morgan—glad to hear the above expression of experience. In June, 1859, had a lady patient with five teeth in which the nerves were exposed, three months advanced in pregnancy, and quite delicate; washed carefully, and applied creosote; capped and filled with Hill's Stopping. About a year after, the lady called again to have the fillings removed and others put in; in three of the teeth the nerves were dead, in the other two a deposit of bone had been thrown out, covering the nerve with a solid cap of bone. Thinks the cases in which the deposit of bone was made had the nerves wounded so as to produce bleeding, and thinks it essential to the deposit of dentine, that the nerve should be wounded.

Dr. Priest has used, in a great many cases, gutta percha and Hill's stopping, and wishes to call out the views of the convention in regard to it.

Dr. W. B. ROBERTS wishes to draw attention to the fact that the greatest amount of decay occurs between the ages of fourteen and twenty. It has been remarked by old practitioners, that to preserve teeth in young patients tin foil is preferred; what he desires to ascertain is, what is the best thing to be used at this age? Has always been a strong advocate of gold and gold only, but has observed many fillings at this age where tin foil had perfectly preserved the teeth, although the filling externally was quite black; on the other hand, some of the finest gold fillings remained perfectly bright, but around the margin the tooth will show a dark ring of decay.

Dr. PRIEST remarked that we should understand that using gutta percha, or other non-conductor, was not done to save gold.

Dr. ROGERS rose to a question of privilege to announce the death of Dr. A. Blaksley, eulogizing his character as a man, a gentleman, and an operator, and his devotion to his profession, and advancement of his profession and this convention, never seeming to grow weary as he advanced in age. With other remarks suited to the occasion, a series of resolutions were offered, adopted, and placed upon the minutes.

Dr. BURRAS followed in some eloquent and appropriate remarks—historic, biographical and eulogistic.

Dr. PERINE, as a pupil of Dr. B., has struggled to suppress his feelings—can do so no longer. He was an honor to the profession—a star of the first magnitude, who needs no monument—his name will go down to posterity with love and admiration.

Dr. ALLPORT could not have been induced to address the chair on any other subject, on this he could not resist. None knew him but to love him; he was devoted to his profession—gentle as a woman—kind as man could be. We all knew him; none knew him but to love him; always ready to assist the young and worthy practitioner up the rugged road of science; none more firmly opposed quackery and empyr cism in every form.

SIXTH SUBJECT—DISEASED DENTINE.

Dr. DODGE having used non-conductors, and mentioning the fact to a gentleman of very high standing, was informed that he found the gutta percha to contract, presented this fact merely for consideration.

Dr. COVELL suggested dissolving and purifying gutta percha, by dissolving in chloroform, as it can thus be made perfectly pure, more contractile, and of any desired thickness.

Dr. ASAY'S experience with gutta percha fillings is not favorable, frequently finding strangulation of the nerve, and no very pleasant odor; found that a quill split open and placed over the exposed nerve, or sensitive dentine, and filling with gold, to result in the happiest effects.

Dr. B. W. FRANKLIN claims that the cause of diseased or sensitive dentine is acid; the remedy is alkali. To prove this apply litmus paper. Take the broad ground, that God designed these organs to remain as long as there is use for them, they never could decay by natural causes. We find, under the free margin of the gum, great sensitiveness, and if we apply litmus, we find acid, and if we apply alkali, we have a removal of the sensitiveness, illustrating by a number of cases; as patients with dyspepsia, with acid secretions; teeth badly decayed, filled twenty years ago, and kept in perfect condition by the daily use of alkali. Usually our whole system of living is false, our system of diet is calculated to destroy instead of promoting health.

Dr. BURRAS—The difference of temperature to which the teeth are subjected is a cause of sensitive dentine, especially in connection with decomposing animal matter, this produces not only sensitiveness, but decay; defects of the teeth are hereditary. Gave cases of hereditary transmission—his *own case.*

Dr. Covell—The acid theory was a favorite one of his, but when he was at the Sandwich Islands he found other things produced decay besides acids.

Dr. Taft read a paper from Dr. Foote. (It is found in this number of the Register.)

Dr. Morgan endorses the sentiment of the paper; thinks that many of the agents used for the treatment of the sensitive dentine are highly injurious, as they destroy the tissue.

Dr. Priest—it does seem we do not go back far enough. He thinks that the chief cause of decay is a deficiency of the organization, more than anything else.

Dr. Atinson thinks that the subject is not well understood. The life force has much to do with the sensitiveness of the teeth, also with the decay. The way to procure good teeth, is to have the *originals good.* If we knew just what caused the disintegration of dentine, we might understand the matter.

On motion, *Resolved,* That the final adjournment be to-morrow, at 1 o'clock, P. M.

Adjourned till 4 o'clock, P. M.

4 o'clock, p. m.

The Convention met according to adjournment.

Minutes read and approved.

*Sixth Subject*—Exposed Pulp.

Dr. Taft considers this a subject that claims the closest consideration of the dentist, for it is only by the strictest conformity to the indications that success can be obtained. There are many circumstances, that will modify the treatment in any given case, for instance, the constitution of the patient; the peculiarities of the constitution; the length of time the nerve has been exposed; the size of the orifice at which it is exposed; and the character of the agents that have been brought in contact with it subsequent to the exposure. With a good constitution, and recent exposure, and by a small orifice, do not hesitate in any case to treat and fill with a view to the preservation of the pulp. It is difficult to give any rule of procedure ; almost every case will require procedure peculiar to and adapted for it. In general terms, the object is to remove all irritating agents, and render the pulp healthy, the latter to be done by topical or constitutional treatment, singly or in connection. Nature alone will often accomplish the work, when the opportunity is offered her. When the restoration of the pulp is accomplished, then imitate nature's arrangement for its future residence as much as possible ; make the chamber of the natural form and size, and place in contact with the nerve, material that will not irritate it, also a non-conductor, then fill the decayed cavity firmly. If a pulp is wounded, it is susceptible of restoration and preservation.

Dr. Westcott—it is with hesitancy and embarrassment that I attempt to speak upon this subject; have not had the success of others; where he exposes the pulp even slightly, it causes an electric shock to him; thinks a living tooth better than a dead one, because the latter is more liable to take on periostitis. What is the prospect, where a pulp is exposed, even in a good case? it is a loss of the nerve. Where we have exposed pulps, and we excavate, we cut away a portion of the cavity that contained the pulp. In no such case can the containing cavity be restored to its natural form, and so the pulp can not be in its natural *condition.* His method of destroying pulps is surgical cutting out with the probe or proper instrument; have not had a single case of abscess by this treatment. Again, sometimes destroys the pulp with arsenic and creosote ; his method is, after cleansing the cavity and exposing the pulp, to place on the exposed point a small roll of cotton moistened with creosote, and with the smallest grain of arsenic; then fill the cavity with a temporary filling of tin; this may remain from two or three days to six months,—if it remains a year, it will do no harm. The pulp will not be decomposed while it is saturated with arsenic. There is no pain from the application.

Dr. DWINELL—asks if arsenic does not pass through the dentine, and thus produce a diseased condition of the periosteum.

Dr. WESTCOTT—thinks that when trouble occurs it is in consequence of the imperfect method of applying it, used but the smallest portion of arsenic. In surgical treatment, abscess never occurs; with arsenic the success is not so good. Fills teeth to which an abscess is attached.

Dr. ATKINSON—thinks an abscess will not occur when the pulp is removed by surgical treatment. When any part becomes unfit for use, the surgeon should cut it clean away, and remove the disintegrated portion. Can show cases of deposition of secondary dentine; there are instances of it in those cases where the teeth are worn down—if there is strangulation of the pulp, it will die. The bone-making principle is periostium, the world over.

Dr. DWINELL—thinks by classifying and selecting the cases, the pulp may be preserved. Where a healthy pulp is exposed in favorable cases, if it is wounded, treat it with camphor, and fill with success. Secondary dentine *is* formed; it begins with the formation of the tooth, and continues till the cavity is filled with dentine.

Destroy the pulp with creosote, clean out the cavity, and drop on a little creosote, then remove the portion cauterized, continue to apply and operate till all the pulp is removed; this is done without pain, and in a short time; thinks it better to leave even a partially decomposed portion of bone on an exposed pulp; have filled teeth with cement, that were soft and semi-gelatinous.

Dr. WETHERBEE—observation teaches the impracticability of saving dental pulps; has attempted it, but failed; where a tooth is filled well and air tight, there will be hydraulic pressure. Destroy the pulp by surgical treatment always when it can be done, and in other cases, with the smallest amount of arsenic ; do not use enough to affect the periosteum. Gave a case, inwhich arsenic was the cause of abscess ; cleaned out the tooth and fang, and filled, and it recovered perfect health ; the deposit of secondary dentine is a very slow process—ten years will sometimes be required ; have never seen a case of secondary dentine formed to protect a nerve.

Dr. Rogers—it is the general rule that the nerve will die, when it is treated as described, and it is wrong to practice on exceptional cases ; the cases are rare in which success is the ultimate result. Do not think that it is safe to leave arsenic in a cavity, as described by Dr. Westcott, though Dr. W. may succeed ; it is a dangerous practice ; the parts will take up the arsenic. In his experience, has never met with any cases of exposed nerve, where the nerve has been saved, often have to rely upon the assertions of the patient ; fill teeth frequently, patient goes away, and we never hear of them again. We take this as a success, when in reality the nerve is dead. A case of this kind came under his observation not long since ; removed the filling, and to his surprise, found no nerve, it was dead and gone, and smelled dreadfully. Would it not have been better to remove the nerve, at first, and filled to the apex of the fang ?

Dr. Westcott—thinks that if the arsenic remained permanently in the cavity, if it is perfectly filled, and the arsenic protected from moisture, no harm will result.

Dr. Roberts—in his own case, has had a nerve treated, and it failed ; is satisfied with nerve treatment.

Adjourned till to-morrow morning, at 9 o'clock.

### FOURTH DAY—9, A. M.

The Convention met according to adjournment, Dr. Buckingham in the chair.

Dr. F. Y. Clark—never attempts to fill a tooth which has its nerve exposed, it will die ; destroy the pulp by the ordinary means, viz: the application of arsenic; when there is abscess, syringe creosote through the fang till it appears at the orifice of discharge.

For restoring the color of teeth, use Darby's prophylactic fluid, remove the decay, dig out the cavity, and pack into it a pledget of cotton, moistened with the fluid. This restores the color of the tooth. The fangs and decayed cavities should be perfectly filled, in order to save the color.

Dr. TEFFT—in destroying a pulp, let the arsenic remain in about twenty-four hours, use about the amount of one-half the size of a small pin's head; is decidedly opposed to filling and drilling into the fang to make an outlet, would not cut off a nerve by drilling into the canal.

Dr. COLBURN—excises the pulps by drilling into the canal, and the excised surfaces will heal up, a cicatrix will be formed.

Dr. ATKINSON—thinks that pulps may be excised, and a cicatrix will be formed, and such a tooth will be perfectly safe.

Dr. MORGAN—in good cases, where a nerve is exposed, even if it is wounded, cleanse out the cavity, and fill, and is certain with good results; has often found secondary dentine in cases where a tooth has been filled over exposed pulps; destroys pulp with arsenious acid in the usual manner. Finds it difficult to fill fangs, very seldom finds a perfect fang filling, fills as well as he can; gave a case of a tooth, in which he filled over an exposed nerve six years before, and recently found the pulp effectually protected by secondary dentine.

Dr. DWINELL—gave a case of a front incisor, in which he exposed the pulp, and wounded it; treated it with camphor; covered it with a plate and filled it, and it was living several years after; thinks that a wounded nerve is as susceptible of restoration to health as a cut finger, have filled many cases, and examined them, and found secondary dentine; in young persons the prospect is best; have practised filling such cases for years with the greatest success. Have a theory in regard to sensitive dentine; the dentine is made up of dental tubuli, the inter tubular membrane or substance becomes irritable, or inflamed, from affection of the nerve.

Restoring the color of teeth; they are discolored by iron from the blood, this being injected into the dentine; the blood is decomposed, the red corpustles are broken up into many fragments, so that it can be forced into the tubuli; have used solvents for iron; chloride of soda and of zinc; the agents as yet applicable for this purpose are quite limited; hope others will be discovered. The field of chemistry is a broad one, and should furnish other agents.

Dr. Rogers—feels impelled to reply to the remarks just made; exceptional cases will not do to base a practice upon; thinks nine-tenths of the pulps treated with a view to save their vitality, even by the best operators, fail. The pulp is encased in secondary dentine very stoutly, as is evidenced in the teeth that are worn down; gave cases going to prove his position; a tooth which he filled for a boy, nerve exposed, but filled, afterwards examined the tooth, and it was not sensitive, cut, and cut into the pulp cavity, found the pulp dead and odorous. Is particular in his method of destroying nerves; over the point of exposure place a layer of wax, and perforate it at the point of the exposure; into that perforation put a small grain of arsenic, and cover over with wax, so as to close effectually.

Dr. Covell—has treated exposed pulps with such results as warrant him in continuing it; thinks if one in eight is saved, that it gives sufficient encouragement to proceed; but his success is better than that.

Dr. Allen—shall go home and resume a practice that he had abandoned several years ago, viz: that of filling over exposed nerves. To destroy nerves, uses arsenic as described; let it remain in about eight hours; when he removes the nerve, he knows he will succeed, but does not know that he will succeed when he tries to save an exposed pulp; but shall now go home and try to save the vitality of pulps again.

Dr. Dwinell—presented an answer to Dr. Rogers in a diagram upon the black board, by which he illustrated the fact that the dentine of the crown is dead after the pulp is destroyed.

Dr. Pease—removes the nerve from a molar tooth ; after removing the pulp, found intense sensitiveness in the canal of one of the fangs, the walls of the canal were exceedingly sensitive ; filled it, and it is yet in the mouth, and serves a valuable purpose ; referred to a case, in which in an inferior molar tooth, the pulp was living after it was cut off at the points of the fang.

Dr. Whitney—for destroying nerves, apply arsenic ; take a small roll of cotton rolled very hard, and moisten with creosote, take the smallest amount of arsenic on the cotton, make the cavity perfectly dry, and apply, close *perfectly ;* let it go, even for weeks ; pain does not occur, then cleanse out and fill.

Dr. Stackpole—has found sensitiveness of the dentine after the pulp is destroyed. It depends much upon the condition, age, etc. of the patient, what our success shall be ; has often filled teeth with exposed nerves, and removed the fillings, and found the nerve dead.

Let us not take the opinions of others, but investigate for ourselves, with the microscope, and everything that we can bring to our aid.

Dr. Pease—extracted the teeth of a gentleman for a set ; attempted to destroy a pulp of an incisor, destroyed part of it, the remainder was thrust with a probe, armed with arsenic, the tooth did not become sore, but there was necrosis of a portion of the alveolus and gum.

Dr. Tefft—uses arsenic, morphine and tannin, removes all substance from the canal, take a grain of the powder, and apply to the nerve, let it remain from twelve to twenty-four hours, then apply tannin and alcohol.

Dr. Meriman—have capped exposed nerves, and filled with success.

Dr. Atkinson read a paper on dental teachings, (which will be published in next number.—Rep.)

The ninth order of business, viz: exhibitions of models, etc., was taken up.

Dr. Sutton presented a case of protrusion of the lower jaw, for the treatment of which he asked the opinion of the members of the Convention. Two or three valuable suggestions were made in regard to it.

Dr. Roberts presented corundum wheels made of vulcanized rubber and emery. They are manufactured by E. A. L. Roberts.

Dr. Franklin presented Dr. Asay's method of attaching teeth to gold or silver plates, with vulcanized rubber instead of solder. Quite a spirited discussion sprung up upon the question, as to whether a man shall exhibit to this Convention materials and implements, where there is a secret in its manipulation or preparation.

On motion, *Resolved*, That Dr. Asay be permitted to exhibit his work, provided he give a full deseription of the process.

On motion of Dr. Rogers, *Resolved*, That the President be authorized to make preparation for the next Convention, or employ some one to do it.

Dr. Franklin—refers to the manner in which a tooth is retained in the socket, referred to a case in which two central incisors which were imported from Paris in alcohol, were inserted into the sockets, to which they became firmly attached. Extracted a second molar, let it remain till he filled the wisdom tooth, and then filled the extracted tooth, and then replaced it in the socket, and it became firmly attached, and is now a good tooth.

Many interesting conversations occurred, to which it is impossible to do justice in a report like this.

The hour of adjournment having arrived, the President made some very impressive remarks, which will certainly not soon be forgotten by those who heard them, after which the Convention adjourned to meet at New Haven, Conn., on the first Tuesday of August, 1861, at 10 o'clock, A. M.

